# Association Between Obesity Severity and Bioelectrical Impedance Analysis-Derived Metabolic Age in Individuals with Type 2 Diabetes Mellitus

**DOI:** 10.3390/metabo16090681

**Published:** 2026-09-16

**Authors:** Hasan Esat Yücel, Tufan Ulcay, Murat Doğan, Saliha Demir, Gizem Çolak, Ruken Oncu, Ebru Ceylan, Muhammed Fırat Aladag, Emre Uguz, Birgül Deniz Doğan, Cahit Ucar

**Affiliations:** 1 Department of Internal Medicine, Faculty of Medicine, Ahi Evran University, 40100 Kirsehir, Turkey; 2 Department of Anatomy, Faculty of Medicine, Ahi Evran University, 40100 Kirsehir, Turkey; tufanulcay@ahievran.edu.tr (T.U.); demir.saliha@ogr.ahievran.edu.tr (S.D.); colak.gizem@ogr.ahievran.edu.tr (G.Ç.); ruken.oncu@ahievran.edu.tr (R.O.); ceylan.ebru@ogr.ahievran.edu.tr (E.C.); emre.uguz@ahievran.edu.tr (E.U.); 3 Department of Family Medicine, Faculty of Medicine, Ahi Evran University, 40100 Kirsehir, Turkey; muratdogan@ahievran.edu.tr (M.D.); birgul.denizdogan@ahievran.edu.tr (B.D.D.); 4 Department of Internal Medicine, Faculty of Medicine Konya, Necmettin Erbakan University, 42030 Konya, Turkey; cahit.ucar@erbakan.edu.tr

**Keywords:** T2DM, metabolic age, obesity, WHtR

## Abstract

Background: Metabolic age (Met-Age), a bioelectrical impedance analysis (BIA)-derived index based on basal metabolic rate (BMR) and body composition, has been associated with cardiometabolic risk. However, its relationship with body mass index (BMI)-defined obesity severity in individuals with type 2 diabetes mellitus (T2DM) remains unclear. This study investigated the association between obesity severity and Met-Age and identified factors associated with Met-Age. Methods: In this retrospective observational study, 683 adults with T2DM were classified into five BMI categories: normal weight, overweight, and class I, II, and III obesity. Anthropometric, laboratory, and BIA-derived body composition data were analyzed using correlation and sex-stratified multivariable linear regression analyses. Results: Although chronological age did not differ significantly across groups, Met-Age increased progressively with increasing obesity severity (*p* < 0.001). The Jonckheere–Terpstra test confirmed a significant ordered increase in Met-Age across BMI categories. BMI, fat mass, and waist-to-height ratio (WHtR) also increased progressively with increasing obesity severity. In the multivariable regression analyses, chronological age and WHtR were positively associated with Met-Age in both sexes, whereas muscle mass showed a weak inverse association with Met-Age only in female participants. No significant correlations were found between Met-Age and fasting blood glucose (FBG) or hemoglobin A1c (HbA1c) levels (all *p* > 0.05). Conclusions: In individuals with T2DM, greater obesity severity was associated with higher BIA-derived Met-Age. WHtR was positively associated with Met-Age in both sexes, whereas muscle mass showed a weak inverse association among female participants. Met-Age may serve as a descriptive index reflecting body composition and metabolic characteristics; however, its incremental clinical value beyond BMI and WHtR remains unproven.

## 1. Introduction

Type 2 diabetes mellitus (T2DM) and obesity are chronic metabolic disorders with rapidly increasing prevalence worldwide, representing major global public health challenges [1]. These conditions are closely interconnected in terms of both etiology and pathophysiology, imposing a substantial economic burden on healthcare systems while contributing to increased morbidity and mortality [2]. The relationship between obesity and T2DM has been extensively documented, with insulin resistance recognized as the central pathophysiological mechanism linking the two disorders [3,4]. In individuals with obesity, particularly those with excess visceral adiposity, chronic low-grade inflammation and adipokine dysregulation play pivotal roles in the development and progression of insulin resistance [5].

Insulin resistance induces a series of biochemical and cellular alterations that impair glucose and lipid metabolism in insulin-sensitive tissues, including the liver, skeletal muscle, and adipose tissue. When pancreatic β-cells are unable to adequately compensate for this metabolic dysfunction, persistent hyperglycemia develops, ultimately leading to T2DM [6,7]. Beyond its effects on glucose homeostasis, chronic hyperglycemia and insulin resistance also adversely influence body composition, promoting increased fat mass, reduced skeletal muscle mass, and the development of sarcopenia, particularly in individuals with poor glycemic control [8,9]. These changes may further exacerbate metabolic dysfunction and contribute to impaired physical function and reduced quality of life [10,11].

Metabolic age (Met-Age) is a parameter derived from bioelectrical impedance analysis (BIA) devices and is estimated primarily based on basal metabolic rate (BMR). An inverse relationship exists between BMR and Met-Age, whereby higher BMR values are associated with lower Met-Age estimates, whereas lower BMR values correspond to higher Met-Age values. Because BMR varies substantially among individuals of the same chronological age, Met-Age has emerged as a more intuitive indicator of metabolic status [12]. Previous studies have demonstrated significant associations between Met-Age and body composition parameters, showing positive correlations with body mass index (BMI), fat mass, visceral adiposity, and body fat percentage [12,13].

An elevated Met-Age may reflect underlying metabolic dysfunction and the presence of unfavorable cardiometabolic conditions. Specifically, a Met-Age exceeding chronological age has been associated with adverse metabolic profiles and an increased risk of cardiovascular disease, whereas a lower Met-Age is generally considered indicative of better metabolic health [14]. Moreover, higher Met-Age values have been strongly associated with metabolic syndrome (MetS) and other cardiometabolic risk phenotypes, significantly increasing the likelihood of MetS [15]. Accordingly, Met-Age has been proposed as a potential predictor of metabolic syndrome, with values exceeding chronological age indicating elevated cardiometabolic risk [16].

However, despite growing evidence regarding the clinical relevance of Met-Age, to our knowledge, no study has investigated its relationship with BMI-defined obesity severity. Therefore, the present study aimed to examine the association between BMI-based obesity severity and Met-Age in individuals with T2DM and to identify factors associated with Met-Age in patients with coexisting obesity and T2DM.

## 2. Materials and Methods

### 2.1. Study Population

**Figure 1:**
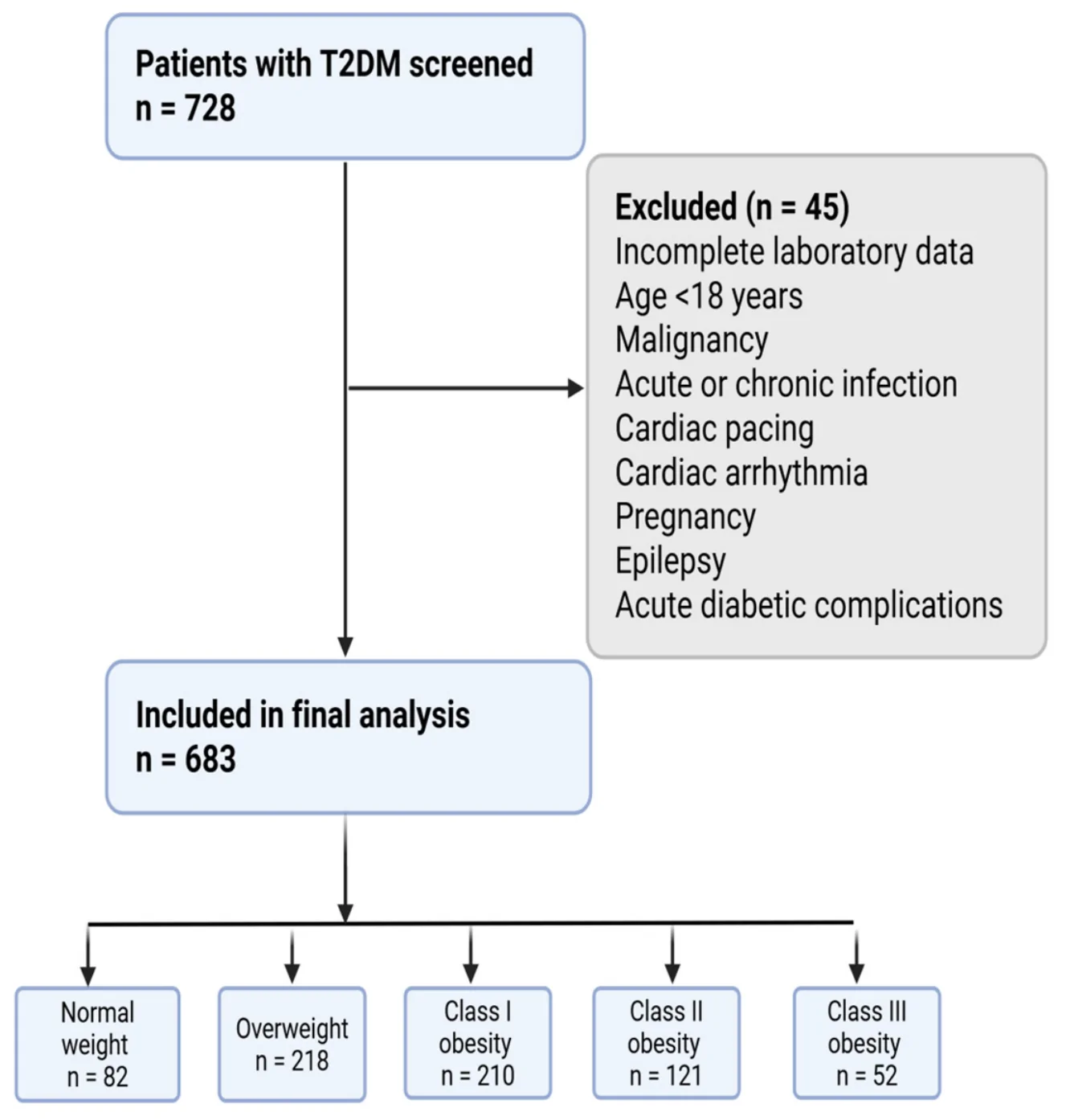
Flow chart of participant selection and classification according to BMI category.

This retrospective observational study included individuals with T2DM who regularly attended the Internal Medicine Outpatient Clinic of Kırşehir Training and Research Hospital between January 2025 and January 2026 and underwent bioelectrical impedance analysis (BIA). Participants were categorized into five groups according to their BMI classification: normal weight, overweight, and class I, II, and III obesity. Individuals aged <18 years and those with malignancy, pregnancy, a cardiac pacemaker, cardiac arrhythmia, acute or chronic infection, or epilepsy were excluded. Individuals with acute diabetic complications, including hypoglycemia, diabetic ketoacidosis, or hyperosmolar hyperglycemic state, were also excluded (Figure 1).

### 2.2. Bioelectrical Impedance Analysis Measurements

BIA measurements were performed using the Tanita MC-780MA analyzer (Tanita Corporation, Tokyo, Japan), which utilizes multi-frequency electrical currents (5, 50, and 250 kHz), as part of routine clinical assessments. In this retrospective study, data from previously performed BIA measurements were obtained from the patients’ medical records. Measurements were performed during the first morning visit following an 8–10-h fasting period. According to the standard measurement protocol, participants were assessed in an upright standing position with their hands and feet in contact with the analyzer’s electrodes and were instructed to remain motionless throughout the procedure.

For body weight assessment, a standard clothing weight of 1 kg was entered into the analyzer and subtracted from the total body weight. Met-Age, muscle mass (kg), and fat mass (kg) were obtained from the BIA measurements performed during routine clinical assessment.

Body composition and basal metabolic rate (BMR) were determined using the Tanita MC-780 multi-frequency BIA device (Tanita Corporation, Tokyo, Japan) with the manufacturer’s integrated software GMON Tanita Pro Health Monitor software, version 3.4.5 (Medizin & Service GmbH, Chemnitz, Germany), which incorporates population-specific reference equations. 

Met-Age was calculated using the manufacturer’s proprietary algorithm based on the estimated BMR relative to age-specific reference values [12]. Because the detailed mathematical formulation of the Met-Age algorithm has not been publicly disclosed by the manufacturer, the independent contribution of variables such as BMI, fat mass, and muscle mass to the final Met-Age calculation cannot be directly determined. Therefore, these variables are assumed to influence Met-Age primarily through their contribution to the estimation of BMR rather than as independently verifiable components of the proprietary algorithm [17,18].

### 2.3. Anthropometric Measurements

Anthropometric measurements were routinely performed in accordance with standardized World Health Organization (WHO) protocols as part of clinical assessment. In this retrospective study, previously recorded anthropometric data were obtained from the patients’ medical records. Waist circumference (WC) was measured using a flexible, non-elastic measuring tape at the midpoint between the lower margin of the last palpable rib and the top of the iliac crest. Measurements were obtained with participants standing upright, with their feet together, at the end of a normal expiration. Hip circumference (HC) was measured at the level of the maximum circumference of the buttocks in a horizontal plane [19].

Height was measured to the nearest 0.1 cm using a BSM 370 stadiometer (Biospace Co., Seoul, Republic of Korea), with participants barefoot. Hip circumference was measured at the widest portion of the hips using a non-elastic measuring tape while participants were standing upright. Waist-to-hip ratio (WHR) and waist-to-height ratio (WHtR) were subsequently calculated from the recorded anthropometric measurements.

Body mass index (BMI) was calculated as body weight divided by height squared (kg/m^2^), and obesity status was classified according to the following BMI criteria [20]:

Underweight: <18.5 kg/m^2^.

Normal weight: 18.5–24.9 kg/m^2^.

Overweight: 25.0–29.9 kg/m^2^.

Class I obesity: 30.0–34.9 kg/m^2^.

Class II obesity: 35.0–39.9 kg/m^2^.

Class III obesity: ≥40.0 kg/m^2^.

### 2.4. Laboratory Measurements

After an 8–10 h overnight fast, venous blood samples were collected into anticoagulant-free gel tubes. Following clot formation, samples were centrifuged at 2000× *g* for 10 min after 30 min of incubation. Fasting plasma glucose (FPG), lipid profile, blood urea nitrogen (BUN), urea, creatinine (cre), aspartate aminotransferase (AST), and alanine aminotransferase (ALT) levels were subsequently measured. Glycated hemoglobin (HbA1c) analyses were performed using whole blood samples collected in K2EDTA tubes. HbA1c levels were measured by high-performance liquid chromatography (Premier Hb9210; Trinity Biotech Co., Wicklow, Ireland).

The estimated glomerular filtration rate (eGFR) was calculated using the race-free CKD-EPI (Chronic Kidney Disease Epidemiology Collaboration) 2021 creatinine equation, in accordance with the current KDIGO 2024 clinical practice guidelines [21].

### 2.5. Statistical Analysis

Statistical analyses were performed using IBM SPSS Statistics for Windows, version 29.0. The distribution of continuous variables was assessed using the Kolmogorov–Smirnov test and visual inspection methods. Continuous variables were presented as mean ± standard deviation or median (interquartile range [IQR]), as appropriate, whereas categorical variables were expressed as frequencies and percentages. Comparisons of continuous variables among the five BMI-defined groups were conducted using the Kruskal–Wallis test, while categorical variables were compared using the Pearson chi-square test.

Because BMI categories represented an ordered severity scale, Jonckheere–Terpstra trend tests were additionally performed to evaluate monotonic trends across BMI categories. For significant Kruskal–Wallis results, pairwise comparisons were interpreted with Bonferroni adjustment where group-level comparisons were required. Because sex distribution differed across BMI categories, an additional general linear model was performed with Met-Age as the dependent variable, BMI category and sex as fixed factors, and chronological age as a covariate. The BMI category-by-sex interaction term was also included to evaluate whether the association between BMI category and Met-Age differed by sex. Estimated marginal means were calculated for BMI categories, and pairwise comparisons were adjusted using the Bonferroni method.

Associations between Met-Age and other clinical and anthropometric parameters were evaluated within each group using Spearman correlation analysis. In addition, the overall obese cohort was further stratified by sex; male and female participants with obesity were compared using the Mann–Whitney U test, and correlation analyses were performed separately for each sex.

Variables significantly correlated with Met-Age in sex-stratified analyses were considered candidate variables for multivariable linear regression. Because BMI, WHtR, WHR, and fat mass are closely interrelated and partly overlap with the BIA-derived Met-Age construct, these variables were not entered simultaneously into the same final model. Multicollinearity was assessed using tolerance and variance inflation factor values. A VIF value < 5 and tolerance > 0.20 were considered to indicate no substantial multicollinearity. Variables with non-significant adjusted associations or substantial conceptual redundancy were not retained in the final models. Because multiple correlation and subgroup analyses were performed, these analyses were considered exploratory. Therefore, interpretation focused on the direction, magnitude, and consistency of associations rather than isolated *p*-values. Cases with missing data for a given variable were excluded only from analyses involving that variable; no imputation was performed. Therefore, the number of observations may vary slightly between analyses involving laboratory parameters.

## 3. Results

Individuals with T2DM were classified into five groups according to BMI: normal weight, overweight, class I obesity, class II obesity, and class III obesity. Sex distribution, anthropometric measurements, BIA parameters, and laboratory findings were compared among groups (Table 1). The proportion of female participants and Met-Age increased significantly with increasing obesity severity (*p* < 0.001). Chronological age was similar across the groups (*p* = 0.191). BMI, WHtR, fat mass, and muscle mass differed significantly across BMI categories. WHR also differed across groups; however, because WHR values showed a compressed distribution in the class III obesity group, WHR-related findings were interpreted cautiously. FBG, HbA1c, total cholesterol, LDL cholesterol (LDL-C), and HDL cholesterol (HDL-C) levels were similar across the groups (all *p* > 0.05). However, triglyceride (TG) levels differed significantly among groups (*p* = 0.045). Among renal function parameters, only creatinine levels decreased with increasing severity of obesity (*p* = 0.009), while no significant differences were observed in the other parameters (all *p* > 0.05).

The Jonckheere–Terpstra test confirmed a significant ordered increase in Met-Age across BMI categories (standardized J-T statistic = 13.273, *p* < 0.001). Significant increasing trends were also observed for BMI, WHtR, and fat mass (all *p* < 0.001). Continuous variables were presented as median (IQR), and categorical variables as n (%). Continuous variables were compared using the Kruskal–Wallis test, and categorical variables were compared using the Pearson chi-square test. Jonckheere–Terpstra trend tests were additionally performed to evaluate monotonic trends across ordered BMI categories. WHR values were available as recorded ratios in the retrospective dataset. The distribution of WHR in the class III obesity group was compressed, most likely because of rounding in the source records; therefore, WHR-related findings were interpreted cautiously, and WHR was not retained in the final multivariable regression models.

Because the sex distribution differed across BMI categories, an additional general linear model was performed to evaluate the association between BMI category and Met-Age after adjustment for chronological age and sex. In this model, BMI category remained significantly associated with Met-Age (F = 427.979, *p* < 0.001, partial η^2^ = 0.718). Chronological age was also strongly associated with Met-Age (F = 8038.830, *p* < 0.001, partial η^2^ = 0.923), whereas the main effect of sex was not significant (*p* = 0.752). The BMI category-by-sex interaction was significant but had a small effect size (F = 5.826, *p* < 0.001, partial η^2^ = 0.034), supporting the use of sex-stratified analyses. Adjusted mean Met-Age increased across BMI categories: 55.69 years in the normal-weight group, 57.47 years in the overweight group, 64.05 years in class I obesity, 69.31 years in class II obesity, and 71.04 years in class III obesity. Bonferroni-adjusted pairwise comparisons showed significant differences between most BMI categories (all *p* < 0.001), except between class II and class III obesity (*p* = 0.129).

Table 2 shows the associations between Met-Age and other study variables. Met-Age was strongly and positively correlated with chronological age in all groups (all *p* < 0.001). BMI showed a weak positive correlation with Met-Age in the overweight and class I obesity groups (both *p* < 0.05). Muscle mass was negatively correlated with Met-Age in the overweight and obesity groups (all *p* < 0.05), whereas fat mass was negatively correlated with Met-Age in the normal-weight group (*p* = 0.019) but positively correlated with Met-Age in the class I obesity group (*p* = 0.021).

WHtR was positively correlated with Met-Age in the overweight and class I obesity groups (both *p* < 0.001), whereas WHR showed no significant association. No significant correlations were found between Met-Age and FBG or HbA1c levels (all *p* > 0.05). Among lipid parameters, only HDL-C showed a positive correlation with Met-Age in the normal-weight and class III obesity groups (both *p* < 0.05). Among renal function markers, urea and BUN levels were positively correlated with Met-Age in all groups (all *p* < 0.001). Creatinine levels were positively correlated with Met-Age in the normal-weight and class I, II, and III obesity groups (all *p* < 0.05), whereas GFR showed a strong negative correlation with Met-Age in all groups (all *p* < 0.001). Among liver enzymes, ALT showed a negative correlation with Met-Age in the overweight and class I–II obesity groups (all *p* < 0.05), whereas AST showed a weak negative correlation only in the class I obesity group (*p* = 0.030). Given the number of subgroup correlations performed, isolated statistically significant associations, particularly those involving lipid and liver enzyme parameters, were considered exploratory findings and should not be interpreted as confirmatory evidence.

The demographic characteristics, chronological age, Met-Age, anthropometric measurements, body composition parameters obtained via BIA, and laboratory variables of all participants with obesity and T2DM were compared by sex in Table 3. There were no significant differences in chronological age or Met-Age between sexes (*p* = 0.884 and *p* = 0.597). Female participants had higher BMI (*p* = 0.006) and fat mass (*p* < 0.001), while male participants had higher muscle mass (*p* < 0.001). WHtR was similar between groups (*p* = 0.885), but WHR was higher in male participants (*p* < 0.001). No sex differences were found in FBG, HbA1c, or TG levels (all *p* > 0.05). T.CHOL, LDL-C, and HDL-C levels were higher in female participants (*p* < 0.001, *p* = 0.002, and *p* < 0.001, respectively). Among renal function parameters, urea and creatinine levels were higher in male participants (*p* = 0.018 and *p* < 0.001), while BUN and GFR showed no significant differences (*p* > 0.05). ALT levels were higher in male participants (*p* = 0.001), whereas AST levels were similar between sexes (*p* = 0.338).

Table 4 shows correlations between Met-Age and other variables in male (n = 96) and female (n = 287) participants with obesity. In both groups, Met-Age was very strongly and positively correlated with chronological age (*p* < 0.001). BMI, fat mass, and WHtR were positively correlated with Met-Age in both sexes (all *p* < 0.001). Muscle mass was negatively correlated with Met-Age in female participants (*p* = 0.001) but not in male participants. WHR was positively correlated with Met-Age in female participants only (*p* = 0.001). No significant correlations were found between Met-Age and FBG or HbA1c in either group (*p* > 0.05). HDL-C showed a weak positive correlation with Met-Age only in male participants (*p* = 0.027). Urea and BUN were positively correlated with Met-Age in both sexes (*p* < 0.001). Cre was positively correlated with Met-Age in female participants but not in male participants. GFR was negatively correlated with Met-Age in both groups (*p* < 0.001). AST and ALT were negatively correlated with Met-Age in male participants (*p* < 0.001), while no significant associations were observed in female participants. These correlation findings were considered exploratory because of the number of subgroup comparisons. Therefore, emphasis was placed on consistent associations, particularly those involving chronological age, WHtR, and selected body composition parameters, rather than isolated significant findings.

Table 5 and Table 6 present the revised sex-stratified multivariable linear regression models for BIA-derived Met-Age among participants with obesity. Variables showing significant correlations in sex-stratified analyses were considered candidate variables. However, because BMI, WHtR, WHR, and fat mass are conceptually and statistically interrelated and partly overlap with the BIA-derived Met-Age construct, these variables were not entered simultaneously into the same final model. Chronological age and WHtR were included in the multivariable regression models based on their strong associations with Met-Age in both sexes. Muscle mass was additionally included in the female-specific model because of its significant correlation with Met-Age.

The overall regression model in male participants (n = 96) was statistically significant (*p* < 0.001) and explained 98.2% of the variance in Met-Age (R^2^ = 0.982; adjusted R^2^ = 0.982) (Table 5). In male participants, chronological age and waist-to-height ratio (WHtR) were strongly and significantly associated with Met-Age. Each one-year increase in chronological age was associated with a 0.932-year increase in Met-Age (B = 0.932; 95% CI: 0.904–0.959; *p* < 0.001). Similarly, an increase in WHtR was significantly associated with an increase in Met-Age (B = 55.341; 95% CI: 50.578–60.105; *p* < 0.001). The VIF value for both variables in the male model was 1.001, indicating no substantial multicollinearity.

In female participants, chronological age and WHtR were also significantly associated with Met-Age. The regression coefficient for chronological age was 0.882 (95% CI: 0.858–0.907; *p* < 0.001). WHtR was positively and strongly associated with Met-Age (B = 44.729; 95% CI: 40.927–48.531; *p* < 0.001). In addition, muscle mass was weakly and negatively associated with Met-Age in female participants (B = −0.062; 95% CI: −0.111 to −0.014; *p* = 0.012). The VIF values in the female model were 1.145 for chronological age, 1.294 for WHtR, and 1.444 for muscle mass, indicating no substantial multicollinearity (Table 6).

Overall, chronological age and WHtR were significantly and positively associated with Met-Age in both male and female participants, whereas muscle mass was significantly and negatively associated with Met-Age only in female participants. The high R^2^ values should be interpreted cautiously because Met-Age is strongly age-dependent and derived from BIA-related body composition and BMR estimates.

To evaluate the incremental contribution of WHtR beyond chronological age, hierarchical regression analyses were performed separately by sex. In male participants, chronological age alone explained 87.9% of the variance in Met-Age, and the addition of WHtR increased the explained variance to 98.2% (ΔR^2^ = 0.103, F change = 532.213, *p* < 0.001). In female participants, chronological age alone explained 86.8% of the variance in Met-Age, and the addition of WHtR increased the explained variance to 95.8% (ΔR^2^ = 0.090, F change = 611.803, *p* < 0.001). These findings indicate that WHtR contributed additional explanatory information beyond chronological age, although the high R^2^ values should be interpreted cautiously because Met-Age is strongly age-dependent and BIA-derived.

## 4. Discussion

Our study provides additional evidence that greater obesity severity among individuals with T2DM of similar chronological age is associated with higher BIA-derived Met-Age values. Chronological age and WHtR were the variables most consistently associated with BIA-derived Met-Age in both sexes among participants with obesity. These findings should be interpreted cautiously. Met-Age is not an independent biological aging biomarker; rather, it is a BIA-derived index based on BMR and body composition-related estimates. Therefore, the observed associations with adiposity-related measures likely reflect, at least in part, the internal structure of the BIA-derived Met-Age algorithm and the close relationship between adiposity, body composition, and BMR. Accordingly, our findings indicate adjusted statistical associations rather than causal or independent biological effects of obesity on aging. Additionally, a weak inverse association between muscle mass and Met-Age was observed among female participants (*p* = 0.012, Table 6).

Previous studies have reported that Met-Age may be associated with anthropometric indicators of cardiometabolic risk. Rodelo et al. reported that Met-Age showed a stronger association with waist-to-height ratio, a recognized marker of cardiovascular risk, compared with chronological age, suggesting that Met-Age may provide complementary information regarding cardiometabolic characteristics beyond chronological age alone [12].

Consistent with our findings, previous studies in individuals with T2DM and MetS have reported that chronological age is associated with Met-Age [12,16], and that WHtR is also positively associated with Met-Age [12]. Nevertheless, future prospective studies are required to determine whether Met-Age provides additional prognostic information beyond conventional anthropometric and metabolic measures.

One of the important contributions of our study is that individuals with obesity exhibited higher Met-Age values compared with those without obesity despite similar chronological ages across BMI groups (Table 1). Furthermore, Met-Age values showed a progressive increase with greater obesity severity. These findings suggest that obesity may represent an important factor associated with the Met-Age profile in individuals with T2DM. However, these results should not be directly interpreted as evidence that obesity accelerates biological aging, as Met-Age is a BIA-derived index based on estimates of body composition and BMR. Therefore, the increase in Met-Age should be considered a potential reflection of obesity-related alterations in body composition and metabolic characteristics. Furthermore, these findings should not be interpreted as evidence that Met-Age offers diagnostic superiority or additional clinical utility compared with conventional anthropometric measures. Future prospective studies should incorporate detailed medication profiles, comprehensive assessments of lifestyle and nutritional status, and reference methods for body composition assessment. Such studies would help determine whether Met-Age provides additional prognostic information regarding clinically meaningful outcomes, including diabetic complications, cardiovascular events, frailty, and mortality.

The finding that WHtR emerged as an important variable associated with Met-Age in individuals with obesity and T2DM is noteworthy (Table 5 and Table 6). The consistency of this association in both sexes suggests that WHtR may have an important relationship with the Met-Age profile. WHtR is a simple and practical anthropometric indicator used to assess abdominal adiposity and body fat distribution, and it has gained increasing attention as a useful parameter in cardiometabolic risk evaluation [22]. Although BMI provides valuable information regarding overall body weight and obesity classification, it has limitations in reflecting fat distribution and, particularly, visceral adiposity [23]. In contrast, WHtR has been reported to show stronger associations with metabolic abnormalities, insulin resistance, and cardiometabolic risk markers, likely due to its ability to better reflect central adiposity. Elevated WHtR values have been associated with obesity-related processes, including increased visceral adipose tissue accumulation, low-grade chronic inflammation, alterations in adipokine balance, and metabolic dysfunction [24]. Therefore, WHtR may serve as a clinically valuable complementary marker, alongside BMI and other body composition measurements, when assessing obesity severity and interpreting Met-Age.

Muscle mass was found to be weakly negatively associated with Met-Age in female participants (Table 6). Previous studies have demonstrated that sarcopenic processes in obesity are associated with increased metabolic risk, and that low lean body mass is linked to cardiometabolic dysfunction [25]. In addition, while lean body mass and skeletal muscle mass appear to exert a protective effect against MetS, fat mass has been shown to increase MetS risk [26]. Given the central role of skeletal muscle in glucose metabolism and energy expenditure, the observed weak inverse association between muscle mass and Met-Age is consistent with current knowledge; however, its clinical or causal significance should be interpreted cautiously.

In the multivariable regression analysis, chronological age and WHtR were positively associated with BIA-derived Met-Age in both sexes (*p* < 0.001, Table 5 and Table 6). However, the high R^2^ values obtained should be interpreted with caution. Because Met-Age is a proprietary index derived from BIA-based body composition and basal metabolic rate (BMR) estimates, a certain degree of algorithmic dependence between the outcome variable and predictors, such as chronological age and anthropometric measurements, cannot be excluded. Consequently, the very high R^2^ values observed in the present regression models may partly reflect the mathematical reconstruction of the device-generated outcome variable rather than independent external or clinical predictive validity. Although collinearity diagnostics indicated acceptable VIF and tolerance values among the retained predictors, these diagnostics evaluate intercorrelations among predictors and do not eliminate dependence between the predictors and the proprietary construction of the outcome.

Nevertheless, the present findings suggest that Met-Age may serve as a complementary descriptive indicator for assessing obesity and cardiometabolic characteristics. However, because Met-Age is a BIA-derived index calculated using the manufacturer’s proprietary algorithm, its clinical applicability and prognostic value require further validation in prospective studies. Notably, recently developed Met-Age models have also suggested that metabolic age may be associated with cardiovascular events and all-cause mortality [12].

The absence of a significant association between glycemic parameters and Met-Age is noteworthy. The lack of correlation of fasting glucose and HbA1c levels with Met-Age suggests that Met-Age may reflect not only glycemic control but also obesity-related indices and broader multidimensional metabolic processes. However, previous studies have reported that increases in HbA1c are associated with corresponding changes in computed tomography (CT)-based biomarkers, including significant increases in visceral adipose tissue (VAT) area, kidney volume, liver volume, and muscle area [27]. Moreover, CT-based body composition analyses have been shown to provide relevant biomarkers associated with metabolic diseases [28]. The difference between these findings and ours may be attributed to the fact that all participants were diagnosed withT2DM, which likely resulted in a relatively homogeneous distribution of glycemic parameters across the study population. Furthermore, our study did not include specific assessments of organ fat accumulation. Instead, body composition was evaluated using BIA, and total body fat mass was derived from this method. Accordingly, the associations between Met-Age and key obesity-related indices, including BMI and WHtR, were more prominently emphasized in our analysis (Table 4).

Associations between renal function parameters and Met-Age are also noteworthy. However, these associations should be interpreted with particular caution because Met-Age is highly age-dependent and showed an extremely strong correlation with chronological age in our study. Moreover, eGFR is itself calculated using age as a component of the CKD-EPI 2021 equation. Therefore, the strong negative association observed between eGFR and Met-Age is likely to be substantially influenced by their shared dependence on chronological age and should not be interpreted as evidence of a direct renal pathophysiological relationship. In this context, the observed association may primarily reflect the age-related structure of Met-Age and the age component incorporated into eGFR rather than an independent effect of renal function (Table 4).

Similarly, the positive associations observed between Met-Age and serum urea and BUN should be interpreted cautiously and regarded as exploratory findings. Given the very strong dependence of Met-Age on chronological age, these associations cannot be considered independent evidence of renal dysfunction without appropriate age-adjusted analyses. The retrospective design of the study further limits causal or pathophysiological interpretation.

Although obesity and T2DM are well established to contribute to renal injury through mechanisms such as chronic inflammation, endothelial dysfunction, and microvascular damage [29,30], the renal associations observed in the present study should not be interpreted as direct evidence of these mechanisms. Rather, they may partly reflect the pronounced age dependence of Met-Age and the incorporation of age into eGFR estimation. Further studies using age-independent or appropriately age-adjusted measures of renal function are needed to determine whether Met-Age has an independent relationship with renal pathophysiology.

Among male participants with obesity, Met-Age was negatively correlated with AST and ALT and positively correlated with HDL-C (AST: r = −0.344, *p* < 0.001; ALT: r = −0.529, *p* < 0.001; HDL-C: r = 0.233, *p* = 0.027; Table 4). Similarly, a positive correlation between Met-Age and HDL-C was also observed in the normal-weight group (Table 2; r = 0.235, *p* = 0.036). Given the large number of correlation and subgroup analyses performed, these associations were considered exploratory and should therefore be interpreted with caution. In particular, the relatively weak positive associations observed between Met-Age and HDL-C should not be regarded as confirmatory evidence, as their direction is contrary to that generally expected for a metabolically protective marker. As no strong biological mechanism could be identified to explain these findings, these associations should be considered potential chance findings arising from the multiple correlation and subgroup analyses performed. Given the exploratory nature of these analyses, no formal false-discovery-rate (FDR) correction was applied for multiple comparisons.

Sex-based analyses revealed that female participants had higher fat mass, whereas male participants had greater muscle mass (*p* < 0.001; Table 1), which is consistent with well-established physiological and hormonal differences reported in the literature. Previous studies have demonstrated that women generally have a higher body fat percentage, while men generally possess greater lean body mass and skeletal muscle mass [31].

This study has several important strengths. First, it addresses a relatively underexplored research question by evaluating the association between obesity severity and BIA-derived Met-Age in individuals with type 2 diabetes mellitus. The well-characterized study population consisting of individuals with T2DM allowed the relationship between obesity-related factors and Met-Age to be investigated within a clinically homogeneous group. Furthermore, the study included a comprehensive assessment of anthropometric measurements, body composition parameters, metabolic indicators, and renal function markers, enabling a multidimensional evaluation of factors associated with Met-Age. In addition, the use of BIA, a practical, non-invasive, and clinically applicable method for assessing body composition-related metabolic characteristics, enhances the potential clinical relevance and applicability of the findings.

The retrospective design of our study limits our ability to establish causal relationships between obesity severity and BIA-derived Met-Age. Several potentially relevant variables were not fully accounted for, including diabetes duration; antidiabetic medication use, such as GLP-1 receptor agonists, SGLT2 inhibitors, insulin, and metformin, physical activity, smoking status, alcohol consumption, nutritional status, comorbidities, hormonal status, including menopausal status in women and androgen status in men, and sleep and sleep-related factors. Because Met-Age is derived from BIA-related body composition and BMR estimates, the absence of these variables may have confounded the observed associations. In particular, the absence of data on antidiabetic medication use may have limited the interpretation of the associations between Met-Age, FBG, and HbA1c and may represent a potential confounding factor. In addition, the Tanita Met-Age algorithm uses manufacturer-specific and population-specific reference equations, and its application to a Turkish T2DM population may introduce systematic bias in absolute Met-Age values. Finally, the incremental clinical value of Met-Age beyond conventional anthropometric measures such as BMI and WHtR remains unproven because no clinical outcomes, such as diabetic complications, cardiovascular events, frailty, or mortality, were evaluated.

Although BIA is a practical, non-invasive, and widely used method for assessing body composition, its accuracy may be limited in individuals with extreme BMI values, particularly those with severe obesity. Consistent with current European Society for Clinical Nutrition and Metabolism (ESPEN) recommendations, BIA-derived measurements should be interpreted cautiously in these populations due to potential limitations in accuracy [32]. Therefore, BIA-derived Met-Age should be considered an integrated index reflecting body composition characteristics and BMR-related parameters rather than a direct marker of biological aging.

## 5. Conclusions

In individuals with T2DM, greater obesity severity was associated with higher BIA-derived Met-Age. Chronological age and WHtR were positively associated with Met-Age in both sexes, whereas muscle mass showed a weak inverse association in women. These findings suggest that Met-Age may reflect differences in body composition and metabolic characteristics associated with obesity. However, given that Met-Age is derived from BIA-based estimates of body composition and BMR using a proprietary algorithm, these findings should not be interpreted as evidence of accelerated biological aging. Furthermore, the present study does not establish that Met-Age provides incremental clinical value or superiority over conventional anthropometric and metabolic measures, such as BMI and WHtR. Further prospective studies incorporating detailed medication and lifestyle data, comprehensive nutritional assessment, and reference methods for body composition are warranted to determine whether Met-Age provides prognostic information beyond established anthropometric and metabolic measures.

## Figures and Tables

**Table 1:** Comparison of Demographic Characteristics, Chronological and Metabolic Age, Anthropometric, BIA, and Laboratory Variables Across Weight and Obesity Status Categories in Individuals with T2DM.

Groups/Variables	Individuals with Normal Weight	Individuals with Overweight	Individuals with Class I Obesity	Individuals with Class II Obesity	Individuals with Class III Obesity	*p* (*p*-Value)
Female participants, n (%)	26 (31.7)	97 (44.5)	146 (69.5)	95 (78.5)	46 (88.5)	<0.001
Chro-Age	60.0 (15.5)	58.5 (15.0)	62.0 (15.0)	60.0 (12.5)	62.0 (18.8)	0.191
Met-Age	55.0 (17.8)	56.5 (12.3)	66.0 (13.5)	70.0 (12.5)	73.5 (16.0)	<0.001
BMI	23.6 (1.98)	27.6 (2.62)	32.1 (2.40)	36.6 (2.10)	42.9 (3.60)	<0.001
Muscle (kg)	49.6 (9.13)	53.95 (15.42)	50.1 (14.50)	52.4 (8.50)	57.8 (9.08)	<0.001
Fat (kg)	13.6 (6.42)	20.6 (5.95)	29.6 (5.65)	37.6 (5.05)	46.35 (7.90)	<0.001
WHtR	0.48 (0.04)	0.54 (0.05)	0.62 (0.04)	0.69 (0.05)	0.77 (0.07)	<0.001
WHR	0.85 (0.08)	0.89 (0.10)	0.89 (0.10)	0.90 (0.10)	1.00 (0.00)	<0.001
FBG	148.0 (83.0)	136.0 (73.0)	133.0 (62.0)	138.0 (59.0)	131.0 (74.0)	0.365
HbA1c	7.6 (2.63)	7.2 (2.13)	7.3 (2.20)	7.5 (2.00)	7.4 (2.00)	0.171
T.CHOL	176.0 (72.1)	198.0 (61.7)	196.1 (69.0)	200.2 (57.4)	190.9 (54.8)	0.241
LDL-C	102.0 (58.0)	115.0 (51.0)	116.0 (61.0)	116.0 (52.0)	116.5 (45.0)	0.865
HDL-C	44.0 (15.0)	46.0 (16.0)	43.0 (16.0)	47.0 (16.0)	48.5 (14.0)	0.217
TG	124.0 (86.0)	147.0 (117.0)	150.0 (127.0)	152.0 (114.0)	151.5 (76.0)	0.045
UREA	30.0 (12.0)	32.0 (12.3)	31.0 (13.0)	31.0 (11.5)	31.0 (17.0)	0.892
BUN	14.0 (5.75)	14.9 (5.90)	14.5 (6.10)	14.5 (5.60)	14.5 (7.93)	0.940
Cre	0.82 (0.25)	0.82 (0.23)	0.77 (0.28)	0.75 (0.18)	0.71 (0.26)	0.009
GFR	93.0 (18.5)	91.0 (19.0)	89.0 (20.0)	90.0 (22.0)	89.0 (30.75)	0.261
AST	17.0 (5.0)	17.0 (9.0)	18.0 (8.0)	17.0 (8.0)	18.5 (9.0)	0.635
ALT	14.0 (10.0)	18.0 (12.0)	18.0 (10.0)	18.0 (12.0)	19.5 (13.0)	0.002

BIA: Bioelectrical Impedance Analysis; Chro-Age: Chronological Age; Met-Age: Metabolic Age; BMI: Body Mass Index; WHtR: waist-to-height ratio; WHR: waist-to-hip ratio; FBG: fasting blood glucose; HbA1c: hemoglobin A1c; LDL-C: low-density lipoprotein cholesterol; HDL-C: high-density lipoprotein cholesterol; TG: triglycerides; BUN: blood urea nitrogen; Cre: creatinine; eGFR: estimated glomerular filtration rate; AST: aspartate aminotransferase; ALT: alanine aminotransferase.

**Table 2:** Correlation Between Met-Age and Study Variables Across Weight and Obesity Categories in Individuals with T2DM.

Variables	Individuals with Normal-Weight (n = 82) r/*p*	Individuals with Overweight (n = 218) r/*p*	Individuals with Class I Obesity (n = 210) r/*p*	Individuals with Class II Obesity (n = 121) r/*p*	Individuals with Class III Obesity (n = 52) r/*p*
Chro-Age	0.960/<0.001	0.933/<0.001	0.950/<0.001	0.983/<0.001	0.990/<0.001
BMI	−0.137/0.220	0.164/0.015	0.137/0.048	0.111/0.227	−0.168/0.233
Muscle (kg)	0.046/0.681	−0.283/<0.001	−0.278/<0.001	−0.192/0.035	−0.308/0.026
Fat (kg)	−0.259/0.019	0.086/0.207	0.159/0.021	0.010/0.910	−0.083/0.560
WHtR	−0.029/0.795	0.273/<0.001	0.339/<0.001	0.165/0.071	−0.039/0.785
WHR	−0.058/0.603	−0.097/0.155	0.049/0.478	−0.092/0.318	−0.017/0.905
FBG	−0.136/0.228	−0.017/0.810	−0.037/0.606	0.035/0.708	0.227/0.110
HbA1c	−0.063/0.587	0.026/0.719	−0.094/0.203	0.065/0.498	0.204/0.156
T.CHOL	0.040/0.723	0.004/0.954	−0.094/0.188	0.016/0.868	−0.058/0.691
LDL-C	0.013/0.909	0.032/0.648	−0.049/0.494	0.033/0.728	−0.064/0.661
HDL-C	0.235/0.036	0.035/0.612	0.042/0.558	0.040/0.672	0.322/0.022
TG	−0.167/0.141	−0.022/0.752	−0.118/0.102	−0.010/0.919	−0.081/0.574
UREA	0.407/<0.001	0.246/<0.001	0.412/<0.001	0.316/<0.001	0.582/<0.001
BUN	0.382/<0.001	0.234/<0.001	0.401/<0.001	0.305/<0.001	0.524/<0.001
Cre	0.323/0.003	0.085/0.224	0.171/0.017	0.339/<0.001	0.426/0.003
GFR	−0.793/<0.001	−0.466/<0.001	−0.543/<0.001	−0.658/<0.001	−0.669/<0.001
AST	0.130/0.248	−0.078/0.264	−0.155/0.030	−0.123/0.188	−0.208/0.148
ALT	−0.201/0.072	−0.208/0.002	−0.266/<0.001	−0.236/0.010	−0.228/0.112

Spearman correlation analysis was used. Values are presented as Spearman’s rho/*p*-value. The number of observations may vary slightly between analyses because of missing laboratory data. BIA: Bioelectrical Impedance Analysis, Chro Age: Chronological Age, Met-Age: Metabolic Age, BMI: Body Mass Index, kg: kilogram, WHtR: Waist-to-height ratio, WHR: Waist-to-hip ratio, FBG: Fasting blood glucose, HbA1C: Hemoglobin A1c, T.CHOL: Total Cholesterol, LDL-C: Low-Density Lipoprotein Cholesterol, HDL-C: High-Density Lipoprotein Cholesterol, TG: Triglycerides, BUN: Blood Urea Nitrogen, Cre: Creatinine, GFR: Glomerular Filtration Rate, AST: Aspartate Aminotransferase, ALT: Alanine Aminotransferase.

**Table 3:** Comparison of Demographic, Anthropometric, BIA, and Laboratory Variables by Sex Among Participants Affected by Obesity and T2DM.

Groups/Variables	Male Participants n = 96	Female Participants n = 287	*p*-Value
Chro-Age	60.0 (15.0)	62.0 (15.0)	0.884
Met-Age	69.0 (14.8)	68.0 (13.0)	0.597
BMI	33.55 (4.70)	35.30 (5.97)	0.006
Muscle (kg)	64.15 (8.52)	50.30 (8.15)	<0.001
Fat (kg)	30.85 (9.85)	35.45 (9.58)	<0.001
WHtR	0.655 (0.08)	0.660 (0.10)	0.885
WHR	1.00 (0.02)	0.90 (0.12)	<0.001
FBG	129.5 (59.0)	134.0 (64.0)	0.729
HbA1c	7.50 (2.18)	7.35 (2.10)	0.549
T.CHOL	177.0 (72.03)	204.0 (58.23)	<0.001
LDL-C	103.5 (56.3)	121.5 (52.3)	0.002
HDL-C	39.0 (12.0)	48.0 (18.0)	<0.001
TG	144.5 (138.0)	154.0 (100.0)	0.767
UREA	32.0 (12.8)	30.0 (13.0)	0.018
BUN	14.9 (6.35)	14.0 (6.10)	0.069
Cre	0.92 (0.24)	0.72 (0.17)	<0.001
GFR	86.0 (22.0)	90.0 (21.25)	0.098
AST	18.0 (10.0)	17.0 (8.0)	0.338
ALT	22.0 (14.0)	17.0 (11.0)	0.001

Continuous variables are presented as median (IQR), and comparisons between sexes were performed using the Mann–Whitney U test. Chro Age: Chronological Age, Met-Age: Metabolic Age, BMI: Body Mass Index, kg: kilogram, WHtR: Waist-to-height ratio, WHR: Waist-to-hip ratio, FBG: Fasting blood glucose, HbA1C: Hemoglobin A1c, T.CHOL: Total Cholesterol, LDL-C: Low-Density Lipoprotein Cholesterol, HDL-C: High-Density Lipoprotein Cholesterol, TG: Triglycerides, BUN: Blood Urea Nitrogen, Cre: Creatinine, GFR: Glomerular Filtration Rate, AST: Aspartate Aminotransferase, ALT: Alanine Aminotransferase.

**Table 4:** Correlation Between Met-Age and Study Variables by Sex Among Participants Affected by Obesity and T2DM.

Groups/Variables	Male Participants Affected by Obesity (n = 96) r/*p*	Female Participants Affected by Obesity (n= 287) r/*p*
Chro-Age	0.927/<0.001	0.920/<0.001
BMI	0.341/<0.001	0.273/<0.001
Muscle (kg)	−0.118/0.250	−0.189/0.001
Fat (kg)	0.318/0.002	0.249/<0.001
WHtR	0.385/<0.001	0.347/<0.001
WHR	0.193/0.059	0.189/0.001
FBG	−0.076/0.473	0.076/0.208
HbA1c	−0.050/0.643	0.028/0.654
T.CHOL	−0.180/0.088	−0.027/0.656
LDL-C	−0.083/0.439	−0.025/0.687
HDL-C	0.233/0.027	0.071/0.242
TG	−0.1527/0.149	−0.066/0.281
UREA	0.372/<0.001	0.411/<0.001
BUN	0.347/<0.001	0.392/<0.001
Cre	0.123/0.243	0.299/<0.001
GFR	−0.494/<0.001	−0.601/<0.001
AST	−0.344/<0.001	−0.065/0.285
ALT	−0.529/<0.001	−0.118/0.052

The relationships between Met Age and other variables were evaluated using Spearman correlation analysis. Chro Age: Chronological Age, Met-Age: Metabolic Age, BMI: Body Mass Index, kg: kilogram, WHtR: Waist-to-height ratio, WHR: Waist-to-hip ratio, FBG: Fasting blood glucose, HbA1C: Hemoglobin A1c, T.CHOL: Total Cholesterol, LDL-C: Low-Density Lipoprotein Cholesterol, HDL-C: High-Density Lipoprotein Cholesterol, TG: Triglycerides, BUN: Blood Urea Nitrogen, Cre: Creatinine, GFR: Glomerular Filtration Rate, AST: Aspartate Aminotransferase, ALT: Alanine Aminotransferase.

**Table 5:** Multivariable linear regression model for variables associated with BIA-derived Met-Age in male participants with obesity and T2DM.

Variables	*B*	% 95 CI	*p*	VIF
Intercept	−26.272	(−29.827–22.717)	<0.001	—
Chronological age	0.932	(0.904–0.959)	<0.001	1.001
WHtR	55.341	(50.578–60.105)	<0.001	1.001
R	0.991			
R^2^	0.982			
Adjusted R^2^	0.982			
Standard error of the estimate	1.4068			
Model *p*-value	<0.001			

Note: Multivariable linear regression analysis was performed using the enter method. Variables were selected from sex-stratified correlation analyses, but highly interrelated adiposity and body composition variables were not entered simultaneously into the same final model. Multicollinearity was assessed using tolerance and variance inflation factor values. B, unstandardized coefficient; CI, confidence interval; VIF, variance inflation factor; WHtR, waist-to-height ratio.

**Table 6:** Multivariable linear regression model for variables associated with BIA-derived Met-Age in female participants with obesity and T2DM.

Variables	*B*	95% CI	*p*	VIF
Intercept	−12.049	(−15.215–8.883)	<0.001	—
Chronological Age	0.882	(0.858–0.907)	<0.001	1.145
WHtR	44.729	(40.927–48.531)	<0.001	1.294
Muscle mass (kg)	−0.062	(−0.111 to −0.014)	0.012	1.444
R	0.979			
R^2^	0.959			
Adjusted R^2^	0.959			
Standard error of the estimate	2.0824			
Model *p*-value	<0.001			

## Data Availability

Data is contained within the article.
